# Multiple cerebral metastases and metastatic aneurysms in patients with left atrial Myxoma: a case report

**DOI:** 10.1186/s12883-019-1474-4

**Published:** 2019-10-23

**Authors:** Yan Wan, Hai Du, Lei Zhang, Shuang Guo, Li Xu, Yuanyuan Li, Hui He, Lian Zhou, Yunping Chen, Ling Mao, Huijuan Jin, Bo Hu

**Affiliations:** 1 Department of Neurology, Union Hospital, Tongji Medical College, Huazhong University of Science and Technology, Wuhan, 430022 China; 2 Department of Neurology, Caidian People Hospital, Wuhan, 430100 China; 3 Department of Pathology, Union Hospital, Tongji Medical College, Huazhong University of Science and Technology, Wuhan, 430022 China; 4 Department of Neuropathology, Beijing Neurosurgical Institute, Beijing, China

**Keywords:** Atrial myxoma, Cerebral metastasis, Metastatic aneurism, Hemorrhage

## Abstract

**Background:**

Cardiac myxoma is the most common benign cardiac tumor. Brain metastases or multiple cerebral aneurysms are extremely rare, especially for the case of both complications. Brain metastases are usually found at the same time or few months after the diagnosis or surgical removal of cardiac myxoma

**Case presentation:**

We describe a case of patient, operated for a cardiac myxoma, who presented multiple central nervous system metastases associated, cerebral aneurysms and subsequent intracerebral hemorrhage

**Conclusions:**

The long-term follow-up of the patients with atrial myxoma even after complete surgical excision is recommended, especially for the patient with central nervous system manifestations before atrial myxoma excision

## Background

Myxomas are the most common tumors of the heart, representing 83% of all primary cardiac tumors with nearly 75% of them arising in the left atrium [1]. Left atrial myxomas are considered highly curable with excellent outcomes in the long-term. Complete resection of cardiac myxoma and its cardiac appendages can cure cardiac myxoma, but incomplete resection, multifocal tumors and embolism caused by tumors are important factors for its recurrence [2].

Cardiac myxoma may cause a wide variety of complications including cardiac obstructive symptoms, systemic embolism and cerebral infarcts [3, 4]. The central nervous system (CNS) is one of the most susceptible areas of embolization most often resulting in ischemic strokes. Cerebral metastatic, intraparenchymal hemorrhage and oncotic aneurysm are uncommon neurological sequelae of atrial myxoma. Since the persistent risk of brain metastases and aneurysms formation, the early diagnosis and intervention is desirable. We reported a case of postoperative patient with left atrial myxoma excision associated with the multiple central nervous system metastases, cerebral hemorrhage and myxomatous aneurysms.

## Case presentation

**Fig. 1:**
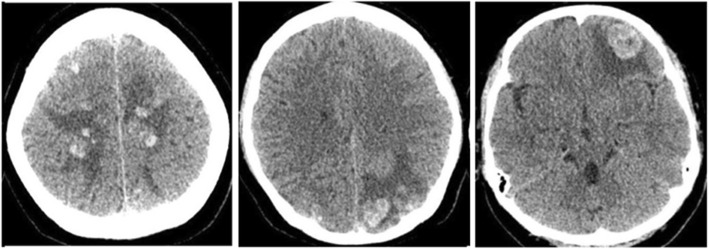
CT brain image shows multiple hemorrhagic lesions surrounded by edema

**Fig. 2:**
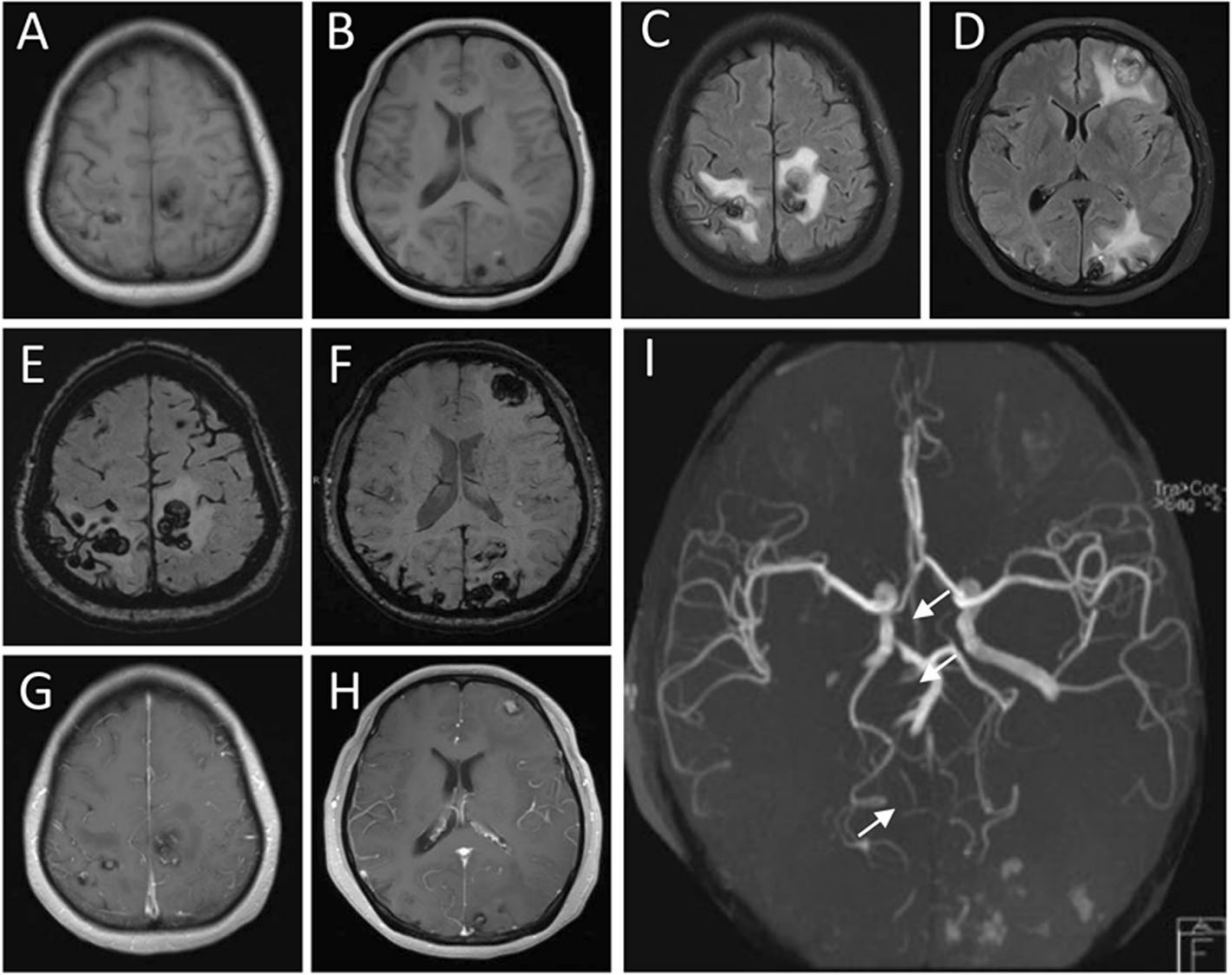
MRI brain images show multiple brain metastases of myxoma, hemorrhage and cerebral aneurysm formation from myxoma. **a**-**b** The axial T1-weighted images. **c**-**d** The axial T2-weighted flair images show multiple nodules or mass shadow. **e**-**f** The axial SWI images shows multiple cerebral hemorrhage. **g**-**h** Magnetic resonance enhanced scan showed heterogeneous reinforcement. **i** The MRA images for aneurysms

**Fig. 3:**
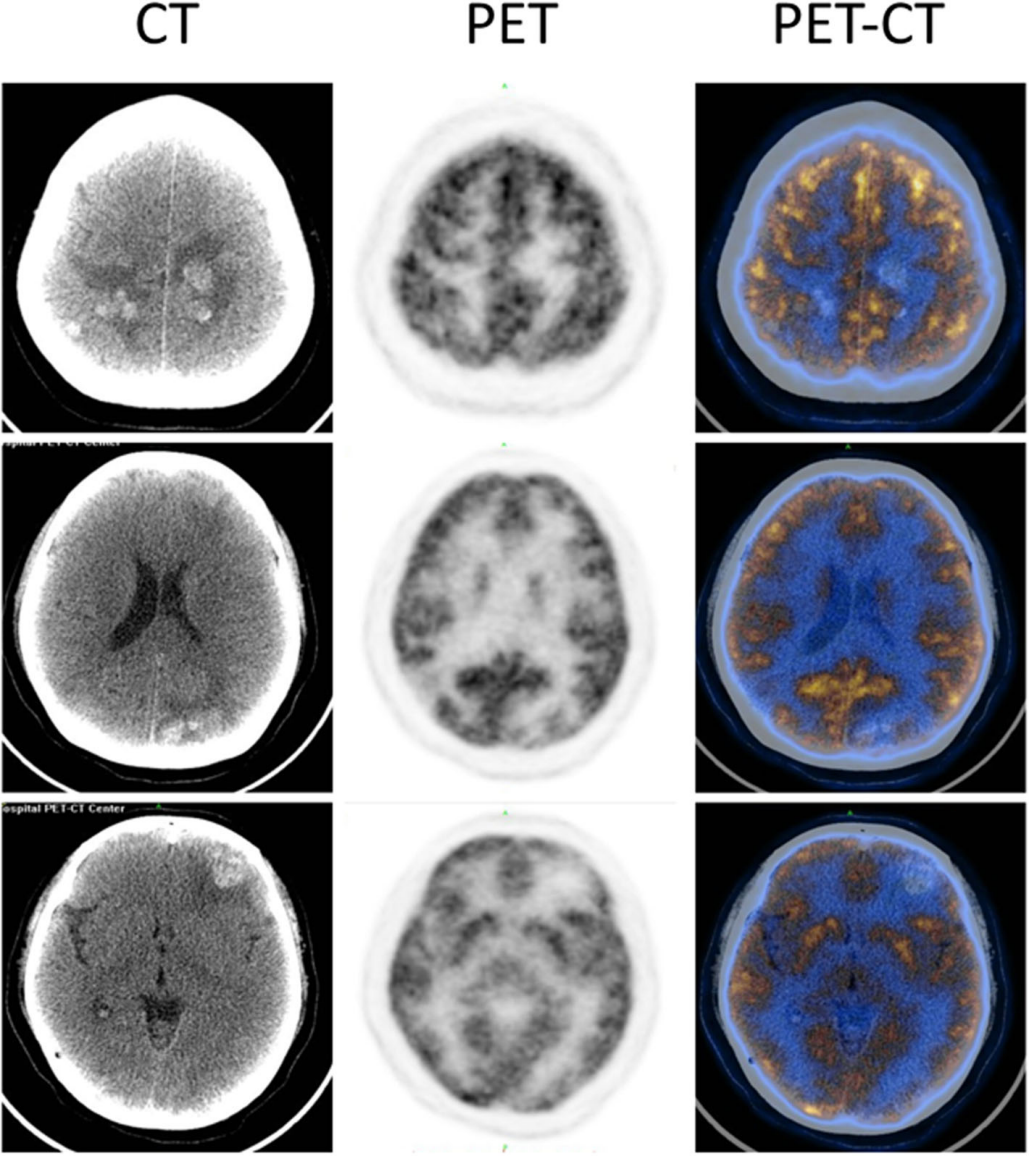
^18^F-FDG PET images show no obvious FDG uptake in the lesions of brain

A 39 years old woman with a background history of left atrial myxoma with total tumor excision done on November 24, 2017, presented to us on June 21, 2018 with progressive headache associated with blurred vision, nausea and vomiting which was lasting for 1 month. There was no history of fever, trauma or seizure. Brain computed tomography (CT) showed heterogeneous hemorrhagic lesions surrounded by edema. The lesions were spread through both hemispheres with the largest one in the left frontal lobe about 2.4*2.2 cm in diameters (Fig. 1). Magnetic resonance imaging including MRI, MRA and SWI confirmed the presence of multiple brain metastases of myxoma and cerebral aneurysm formation from myxoma involving the bilateral anterior cerebral artery (ACA) and middle cerebral artery (MCA), right posterior cerebral artery (PCA) and superior cerebellar artery (SCA) (Fig. 2). Subsequent ^18^F-FDG PET-CT was performed to exclude malignancy (Fig. 3). The echocardiographic showed no recurrence of the atrial myxoma.

**Fig. 4:**
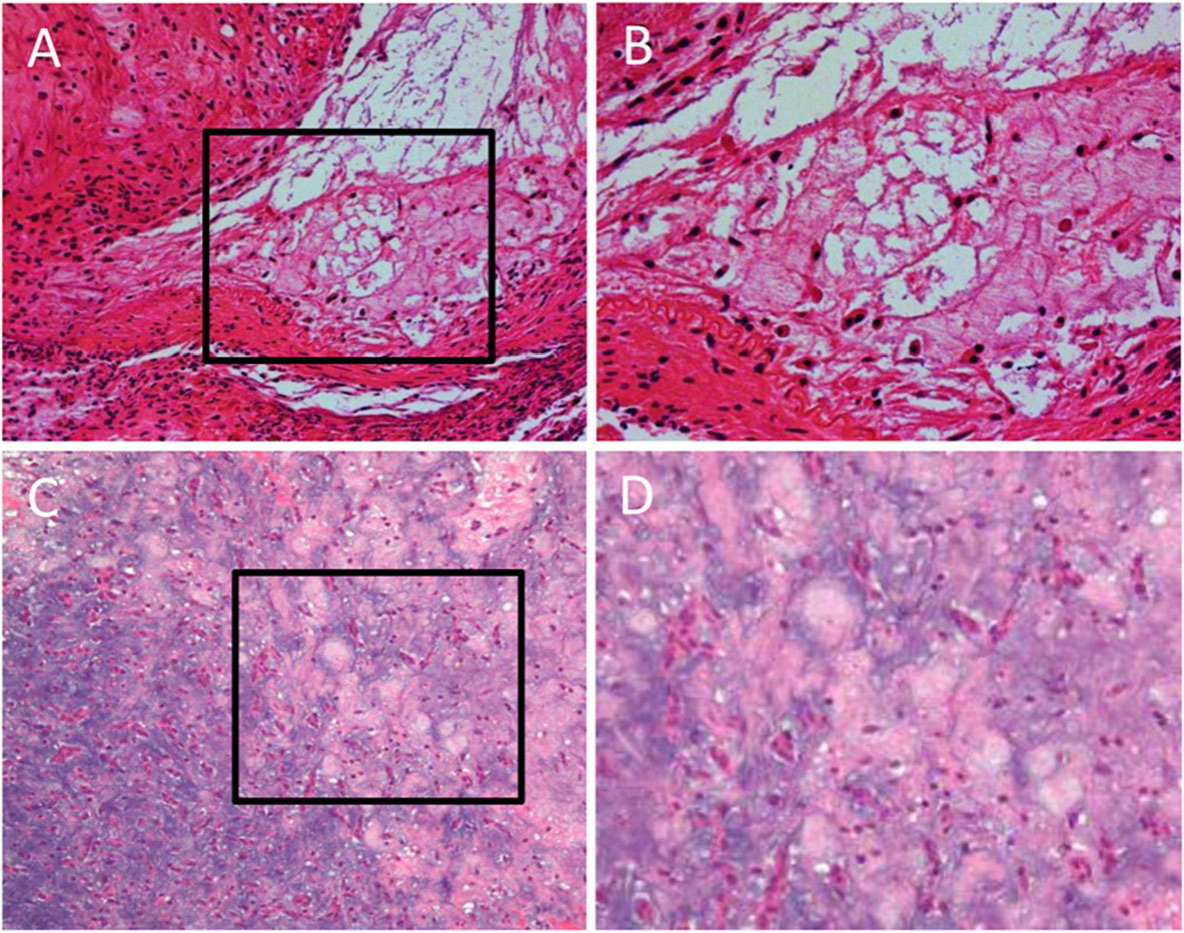
Photomicrographs of the excised left frontal tissue section and cardiac tissue revealing the presence of myxoma cells. H & E. **a** Low-power magnification of brain tissue (× 20). **b** High-power magnification of the area in A surrounded by the black line (× 40). **c** Low-power magnification (× 20) of cardiac tissue. **d** High-power magnification of the area in C surrounded by the black line (× 40)

On July 12, 2018, the patient was admitted to the Beijing Tiantan Hospital for treatment. A left frontotemporal craniotomy was performed. The left ACA arterial aneurysms, which were responsible for intracranial hemorrhage in the left frontal lobe lesion, were neurosurgical clipping and a little bit of the frontal tissue (4*4*8 mm) was excised for histological examination. For safety of the patient, other lesion sites involving the right ACA and the bilateral MCA, right PCA and SCA were not treated by surgery. And they found myxoma cells in the brian tissue by histological examination confirming the cerebral metastases despite 7 months having passed since the cardiac myxoma was resected. Hematoxylin and eosin staining of both tissue disclosed typical atrial myxoma cells, embedded in a loose myxoid matrix in the resected tissue (Fig. 4).

**Fig. 5:**
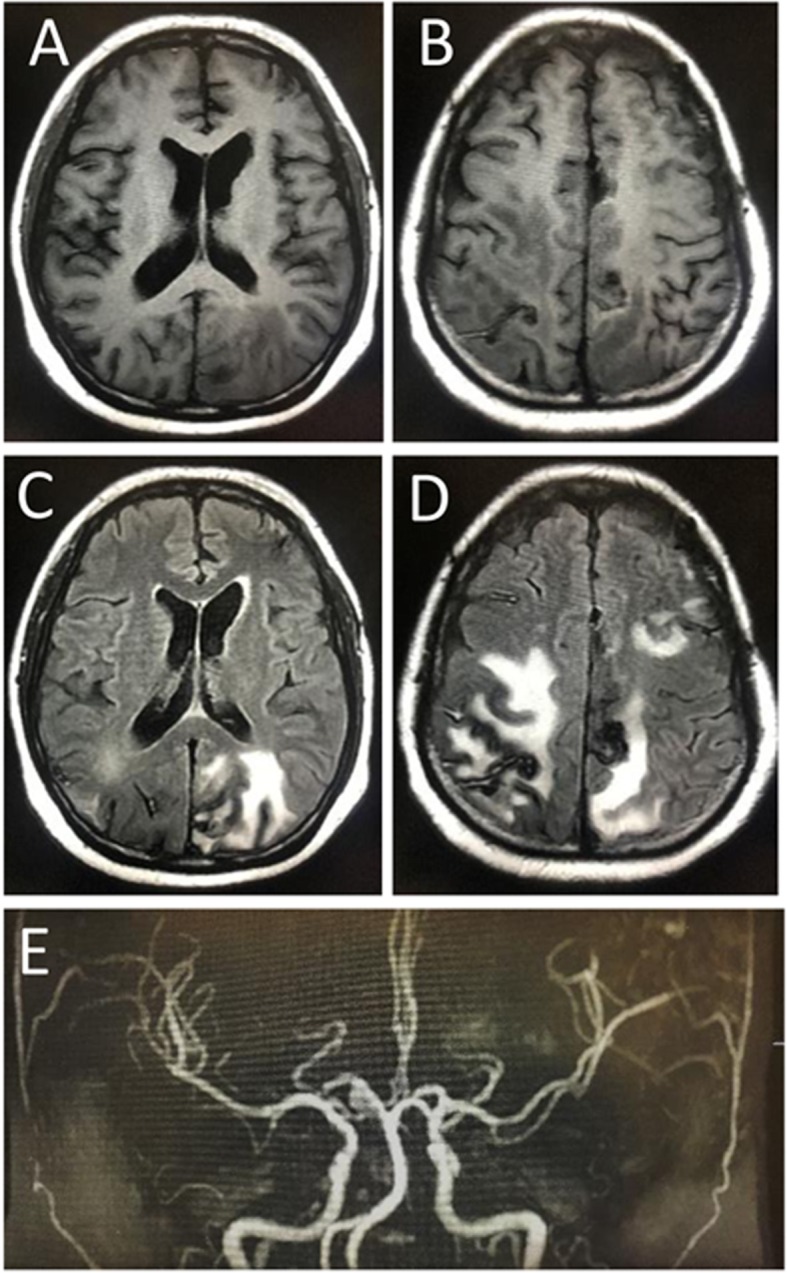
MRI brain images from follow-up examination. **a-b** The axial T1-weighted images. **c-d** The axial T2-weighted flair images show multiple nodules or mass shadow. **e** The MRA image for aneurysms

After the surgery, the patient received antiepileptic, antiemetic and rehydration treatment and other symptomatic treatments. Her headache, blurred vision, nausea and vomiting disappeared gradually. Ten days later, she had a very good recovery and discharged from the hospital. After then, she received antiplatelet treatment and a follow-up observation. In May, 2019, she received reexamination of magnetic resonance imaging including MRI and MRA. Results showed that the brain lesions became smaller, but edema around them became larger than before and the metastatic aneurysms rarely changed (Fig. 5). However, no headache, nausea, vomiting, and other neurological symptoms appeared, so she kept the antiplatelet treatment and follow-up observations.

## Discussion and conclusion

Cardiac myxoma represents mostly benign, slowly proliferating tumor which are believed to derive from multipotent mesenchymal cells of the endocardium [5]. It may present with many clinical syndromes outside the cardiac events. It was reported that about 20–30% of patients with atrial myxoma had neurological complications [3, 6, 7]. The CNS manifestations include progressive headache, nausea or vomiting due to the increase of intracranial pressure, limbs or body weakness or numbness and new onset of seizure [6]. In the majority of cases such complications appear in rather young patients and are usually caused by cerebral embolism and subsequent cerebral infarcts [7, 8]. Cerebral metastatic, aneurysm formation and subsequent subarachnoid or intracerebral hemorrhage are rare but well-known complications of atrial myxoma in adults [9]. Furthermore, aneurysms secondary to myxoma can be a delayed presentation and can be found before resection of myxoma or even after resection of myxoma [10, 11], as in this case. So it is necessary to have a screening with regard to cerebral complications in patients with atrial myxoma. Imaging studies as CT, MRI and cerebral angiography should be performed in every patient with atrial myxoma to detect aneurysms and cerebral lesion. The echocardiography is also suggested for every patient presenting a cerebral infarction with aneurysm [12].

Brain metastases are usually found at the same time or few months after the diagnosis of cardiac myxoma. Other systemic metastases of cardiac myxoma concomitant with the brain have been reported in kidney, pancreas, bone, muscles, skin and stomach [4, 13]. All of these metastases can be explained by the dislodgement of the friable and spongy component of the atrial myxoma into the systemic circulation [14]. The myxomatous aneurysm formation process remains unclear. Three causes of cerebral aneurysm formation have been proposed [14, 15]. One is the vascular damage hypothesis. They postulated that large myxomatous emboli cause vascular occlusion and perivascular damage with subsequent scarring and pseudoaneurysm formation. The other cause is the cerebral vessels are infiltrated by myxoma cells via vasa vasorum causing the destruction of arterial wall architecture, which is similar to the mechanism of mycotic aneurysms. The last cause that has been suggested is the neoplastic process. Several authors have observed myxomatous cells in the aneurysmal vessel. They hypothesized the myxomatous cells could remain viable and penetrate intact or damaged endothelium at the site of final lodgement, with subintimal growth, destruction of the wall, and fibroblastic proliferation. This neoplastic process was accompanied by connective tissue proliferation and a mild inflammatory reaction, and neovascularization in the affected arterial wall may lead to minor bleeding.

Cardiac surgery is the method of choice in treating myxoma [16]. And once the diagnosis is established, surgery should be performed promptly to reduce the possibility of embolic complications or sudden death. For the surgery, three important points should be noted to prevent recurrence: 1) multifocal tumors in heart chambers should not be omitted during operation [17]; 2) cardiac appendages should be removed, sutured and repaired if necessary [18–21]; 3) tumors must be avoided from fragmentation and embolization [19].

Nevertheless, there are no precise guidelines for possible treatment procedures of neurological complications [5, 14]. The use of anticoagulants or antiplatelet agents should be considered in order to prevent further emboli. Surgery is appropriated in cases with few isolated aneurysm or brain metastases or when one of the lesions is life threatening. Moreover, chemotherapy with doxorubicin or ifosfamide with or without radiotherapy of the whole brain could be administrated to patients with multiple brain metastases in order to obtain a longer period of time without recurrence [22]. Furthermore, it is noted that most of the aneurysms are fusiform in shape, and clipping or coiling treatment can be limited. Though there are successful cases of endovascular coiling for enlarging aneurysms, overall evidence documenting stability of cerebral aneurysms over several years gives support to conservative treatment management options [12, 23–25].

Thus, long term follow-up of the patients with atrial myxoma even after complete surgical excision is recommended [16]. Thorough explanation of the potential risks of recurrence of the cardiac tumor, the late development and enlargement of cerebral lesions are necessary. Vigorous work-up must be pursued if the patient again becomes symptomatic or develops new central nervous system manifestations.

## Data Availability

The datasets used or presented during this study are available from the corresponding author on reasonable request.

## Abbreviations

ACA: Anterior cerebral artery
CNS: Central nervous system
CT: Computed tomography
MCA: Middle cerebral artery
PCA: Right posterior cerebral artery
SCA: Superior cerebellar artery
